# Antinociceptive efficacy of lacosamide in the monosodium iodoacetate rat model for osteoarthritis pain

**DOI:** 10.1186/ar2121

**Published:** 2007-02-06

**Authors:** Bettina Beyreuther, Noëlle Callizot, Thomas Stöhr

**Affiliations:** 1 Schwarz BioSciences GmbH, Department of Pharmacology/Toxicology, Alfred-Nobel-Str., 40789 Monheim, Germany; 2 Neurofit, Parc d'Innovation, Rue J Sapidus, 67400 Illkirch, France

## Abstract

The etiology of osteoarthritis is multifactorial, with inflammatory, metabolic, and mechanical causes. Pain in osteoarthritis is initiated by mild intra-articular inflammation and degeneration of articular cartilage and subchondral bone. The principle of treatment with acetaminophen or non-steroidal anti-inflammatory drugs is to reduce pain and improve joint function. Recently, animal models for osteoarthritic pain behavior have been established. The most frequently used rat model for analyzing properties of drugs on the pathology of osteoarthritis is the injection of the metabolic inhibitor monosodium iodoacetate into the joint, which inhibits the activity of glyceraldehyde-3-phosphate dehydrogenase in chondrocytes. Here, we characterize the effect on pain behavior of lacosamide, a member of a family of functionalized amino acids that are analogues of endogenous amino acids and D-serine, in the monosodium iodoacetate rat model for osteoarthritis in comparison to diclofenac and morphine. Lacosamide (3, 10, and 30 mg/kg) was able to reduce secondary mechanical allodynia and hyperalgesia similarly to morphine (3 mg/kg). In contrast, diclofenac (30 mg/kg) was only effective in reducing secondary mechanical hyperalgesia. During the first week, pain is induced mainly by inflammation in the iodoacetate model, but afterwards inflammation plays only a minor role in pain. Lacosamide was able to inhibit pain at days 3, 7 and 14 after induction of arthritis. This shows that lacosamide is able to reduce pain behavior induced by multiple mechanisms in animals.

## Introduction

Osteoarthritis is the most common form of joint disease and a leading cause of chronic pain and disability [1]. Its prevalence after age 65 is about 60% in men and 70% in women [2]. Osteoarthritis is primarily noted in hands, knees, hips, and spine and involves degeneration of articular cartilage as well as changes to the subchondral bone, variable degrees of mild synovitis and thickening of the joint capsule. Characteristic osteoarthritis features are seen in X-ray examination of joints. However, the pathophysiology behind the structural changes associated with this disease is complex and the link to pain is poorly understood [3].

Pain is the predominant clinical feature, yet therapy is ineffective for many patients. Drugs that modify disease progression represent the ultimate goal of treatment but are not clinically available. Treatment is currently based on symptomatic relief of pain and inflammation associated with osteoarthritis to increase joint function. Non-steroidal anti-inflammatory drugs (NSAIDs) and acetaminophen are the most widely used drugs but cyclooxygenase 2 inhibitors, steroids, and opiates are prescribed as well [4].

Several arthritis animal models have been established [5]. One of the best characterized rat models for analyzing properties of drugs on the pathology of osteoarthritis is the injection of the metabolic inhibitor monosodium iodoacetate (MIA) into the joint, which inhibits the activity of glyceraldehyde-3-phosphate dehydrogenase in chondrocytes, resulting in disruption of glycolysis and eventually in cell death [6,7]. The progressive loss of chondrocytes results in histological and morphological changes of the articular cartilage, closely resembling those seen in osteoarthritis patients. However, analysis of pain behavior has only recently been established in the iodoacetate model [8-11]. In a previous study the anticonvulsant gabapentin was able to reduce secondary mechanical allodynia but not hyperalgesia in the iodoacetate rat model [12]. Thus, in order to improve the knowledge on basic pathophysiological mechanisms of osteoarthritis pain, the characterization of animal osteoarthritis pain models and treatment regimens need to be improved.

This study aimed to identify the antinociceptive effect of lacosamide (SPM 927, previously referred to as harkoseride (R)-2-acetamido-N-benzyl-3-methoxypropionamide, ADD 234037), a member of a series of functionalized amino acids that were specifically synthesized as anticonvulsive drug candidates, in the iodoacetate rat model in comparison to morphine (μ-opioid receptor agonist) and diclofenac (NSAID). Lacosamide has been shown to be active in animal models for inflammatory pain [13], for diabetic neuropathic pain [14], and in a small-scale controlled trial of lacosamide in patients with neuropathic pain [15]. Currently, lacosamide is in phase 3 clinical development for neuropathic pain and epilepsy.

## Materials and methods

### Animals

All experiments were carried out in accordance with the guidelines of the committee for Research and Ethical Issues of The International Association for the Study of Pain^® ^(IASP) and in accordance with the institutional guidelines.

Male Wistar rats (Janvier, Le Genest Saint Isle, France) weighing 170 to 200 g at the start of the experiments were used. The animals were group-housed (3 animals per cage) in a room with controlled temperature (21 to 22°C), and a reversed light-dark cycle (12 h/12 h), and they had access to food and water *ad libitum*.

### Development of osteoarthritis in the rat

Osteoarthritis was induced by intra-articular injection of 50 μl of 3 mg MIA (Sigma, Lyon, France) through the patellar ligament of the right knee. Control rats were injected with an equivalent volume of saline. Up to five days after the MIA injection a substantial inflammation of synovial joints was observed in this model. The general health of the animals was monitored. No signs of distress were seen.

### Histology

In a satellite group, histological changes were assessed to confirm the success of the induction of the arthritis of the rat knee joints before and on days 3 and 14 after MIA injection. Knee joints were removed and fixed overnight in 10% formalin and subsequently decalcified by 10% formic acid for 72 h before being embedded in paraffin. Frontal sections (10 μm thick) of the medial aspect of the rat knee joints were prepared every 250 μm. Hematoxylin and eosin staining was carried out to assess the extent of inflammatory infiltrates in the joints and surrounding tissues and safranin-O fast green staining was done to measure the degeneration of cartilage.

### Evaluation of the effect of lacosamide on nociception in vehicle treated animals

Before testing the effect of lacosamide on MIA-injected animals, lacosamide was tested in a control experiment for its possible autonomic influence on pain behavior. Rats were tested in the von Frey test in comparison to vehicle rats 30 minutes post-intraperitoneal treatment with 8, 16, and 32 mg/kg lacosamide.

### Evaluation of the effect of compounds on nociception

In the first round of experiments, the MIA-treated rats were randomized to 6 experimental groups (12 animals per group), which received the following treatments on the days of pain assessment (days 3, 7 and 14 post-MIA treatment): oral gavage of saline (vehicle); oral gavage of 3, 10 or 30 mg/kg lacosamide; subcutaneous injection of 3 mg/kg morphine. Diclofenac (30 mg/kg, subcutaneous) was tested in a separate experiment but by the same scientists under the same conditions at about the same time. The non-MIA-treated control group (control) received oral gavage of saline 45 minutes prior to the pain assessment. Lacosamide, diclofenac and morphine were injected 45 to 60 minutes prior to the implementation of behavioral tests. All rats were trained on the endpoints during the week before the experiment started. The animals were allowed to acclimate to the test room 15 minutes prior the behavioral test. In the von Frey test, fibers were applied after approximately 10 minutes habituation. Each group was examined blind.

### Evaluation of secondary tactile allodynia and mechanical hyperalgesia

For testing tactile secondary allodynia rats were placed on a metallic grid floor. The nociceptive testing was done by inserting the von Frey filament (Bioseb, Chaville, France) through the grid floor and applying it to the plantar surface of the hind paw. A trial consisted of several applications of the different von Frey filaments, always to the same region of the paw at a frequency of 1 to 1.5 s. The von Frey filaments applied were from 10 g to 100 g. As soon as the animal removed its hind paw, the test was stopped and the filament number was recorded to represent the paw withdrawal threshold.

In the secondary mechanical hyperalgesia test, nociceptive flexion reflexes were quantified using the Randall-Selitto paw pressure device (Bioseb, Chaville, France), which applies a linearly increasing mechanical force to the dorsum of the rat's hind paw. The paw withdrawal threshold was defined as the force at which the rat withdrew its paw. The cut-off pressure was set to 250 g.

### Drugs

Lacosamide was synthesized at Schwarz BioSciences GmbH and morphine sulfate was obtained from Francopia (Paris, France). The drugs were dissolved in saline. MIA and diclofenac were purchased from Sigma (Lyon, France). Drug administration was made in a volume of 1 ml/kg.

### Data analyses and statistics

Comparisons of groups of behavioral data at each individual time point were conducted using analysis of variance (ANOVA) followed by *post hoc *analysis (Dunnett's test). Results are presented as means and standard error of the mean (SEM) and analyses revealing *P *values ≤ 0.05 were deemed to be statistically significant.

## Results

### Histological analysis

**Figure 1:**
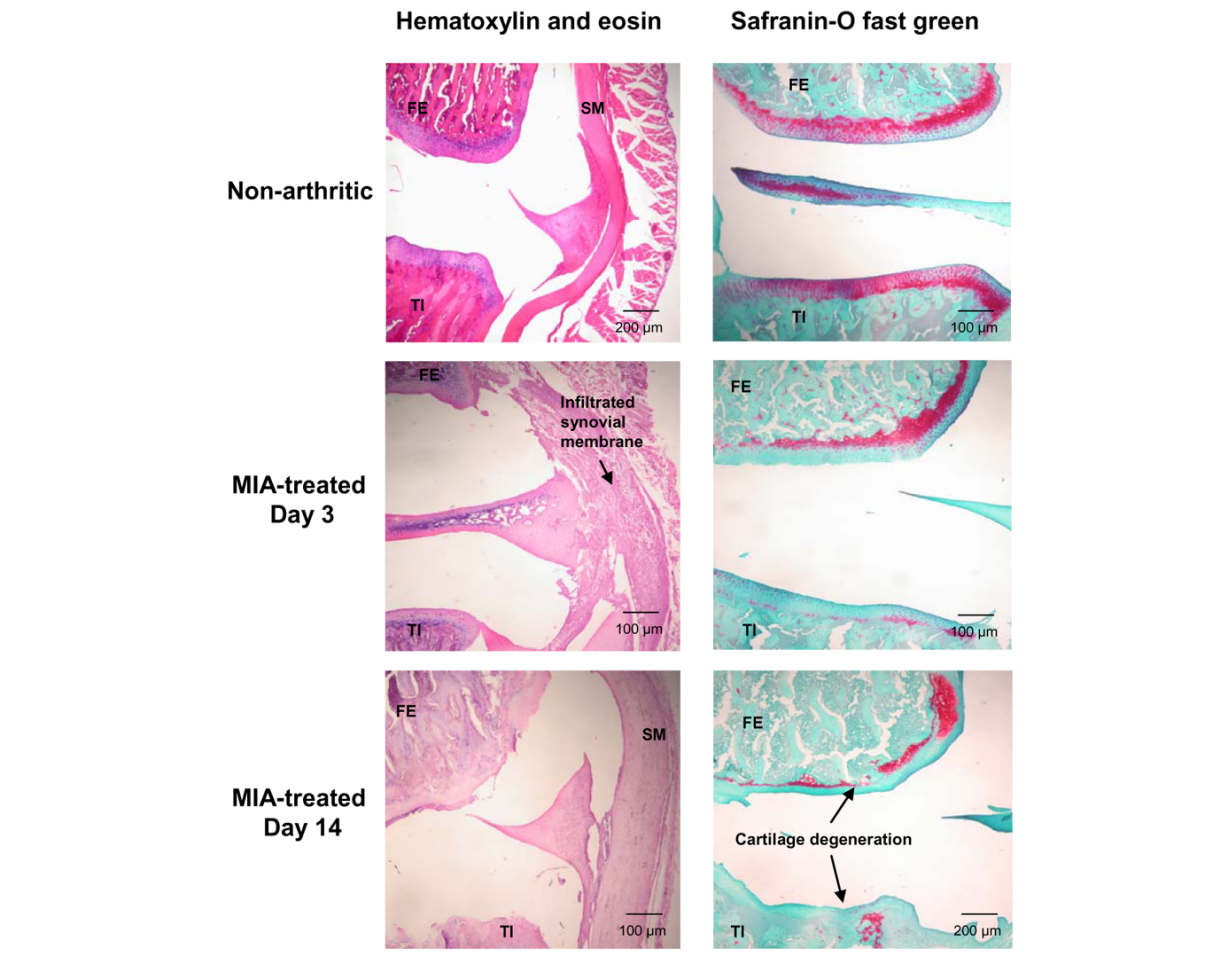
Histological analysis of synovial tissue and articular cartilage pre- and post-monosodium iodoacetate (MIA) injection. Time course of histological changes of the rat knee before and days 3 and 14 after MIA injection. Left sections of the medial aspect of rat knee joints were stained with hematoxylin and eosin, right sections with safranin-O fast green. In non-arthritic rats no abnormality is present. On day 3 after MIA treatment the hematoxylin and eosin stained sections show significant expansion of the synovial membrane with a large amount of cellular infiltrate. No inflammation can be seen on day 14 post-MIA injection. The sections stained with safranin-O fast green reflect major cartilage degeneration on day 14 post-MIA injection. SM, synovial membrane; FE, femur; TI, tibia.

At day 3 a substantial initial inflammatory response was visible in the hematoxylin and eosin staining of the rat knee joints (Figure 1). This inflammation was characterized by an expansion of the synovial membrane, most likely caused by proteinaceous oedema fluid and fibrin with infiltrating macrophages, neutrophils, plasma cells and lymphocytes. As demonstrated in the sections stained with safranin-O fast green, the cartilage was still intact. On day 14 proteoglycan loss was seen throughout the depth of the cartilage. The synovial membrane looked normal and contained no inflammatory cells.

### Effect of drug treatment on secondary tactile allodynia

**Figure 2:**
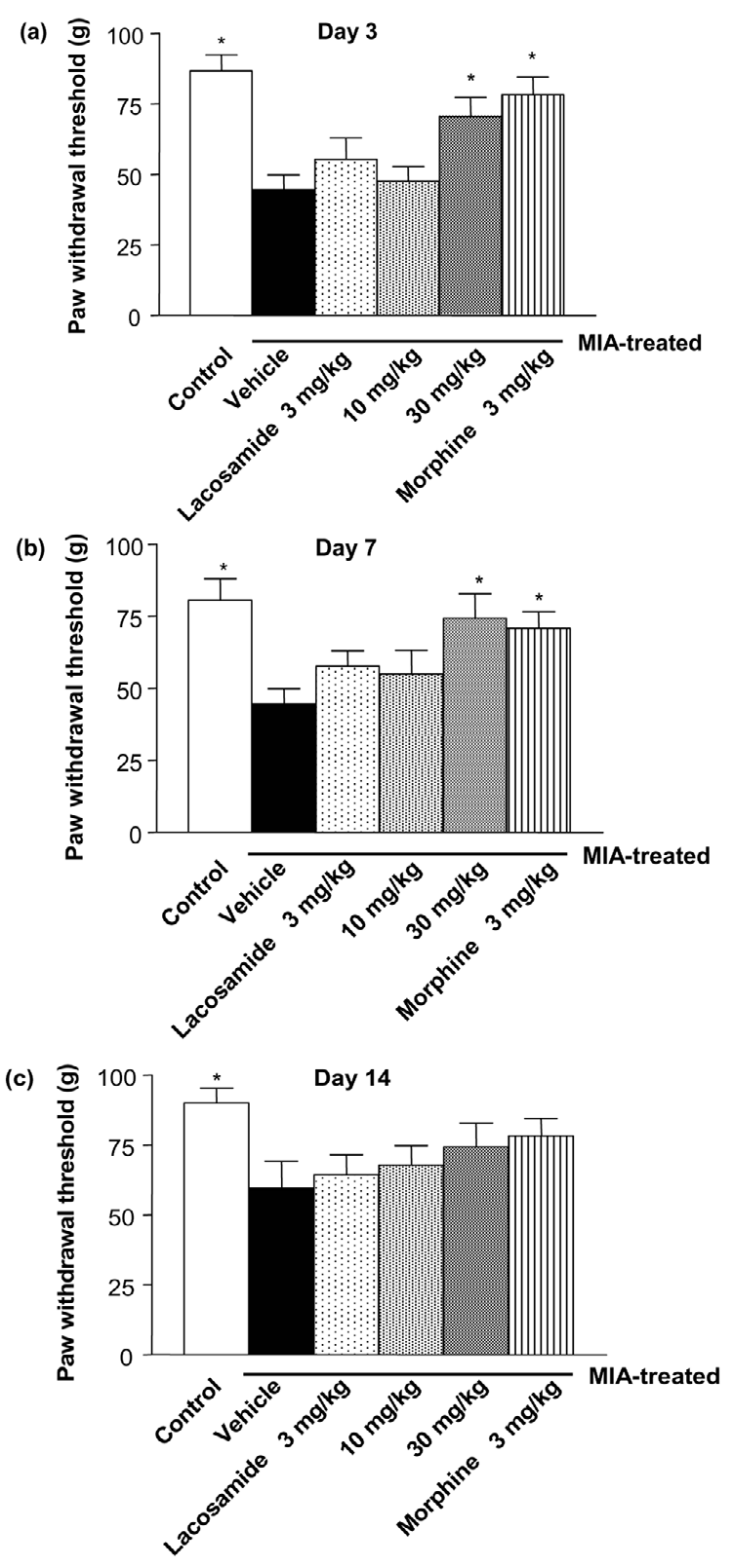
Effect of lacosamide and morphine on tactile allodynia after monosodium iodoacetate (MIA) injection. Effect of lacosamide and morphine on paw withdrawal threshold in the von Frey filament test in iodoacetate-treated animals on **(a) **day 3, **(b) **day 7 and **(c) **day 14 after arthritis induction. Rats were tested 45 to 60 minutes post-drug. Data from 12 animals/group are presented as mean ± SEM. *P < 0.05 Dunnett's test versus MIA/vehicle treated animals.

**Figure 3:**
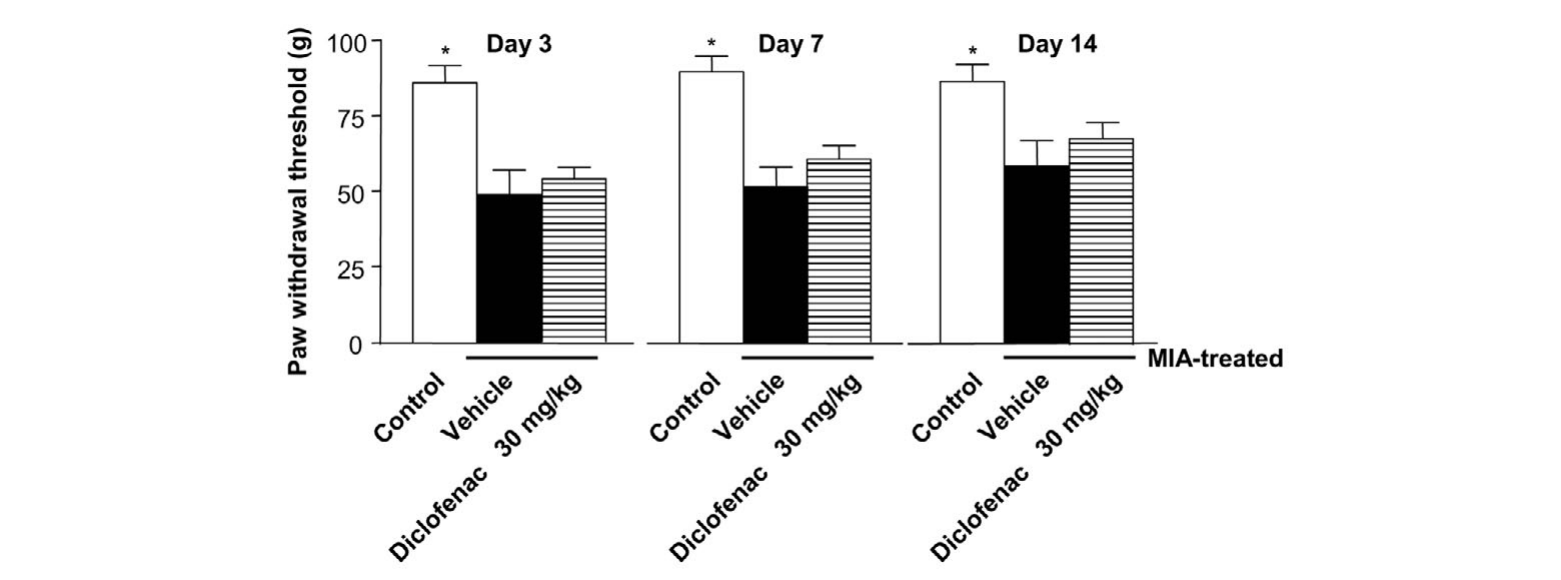
Effect of diclofenac on tactile allodynia after monosodium iodoacetate (MIA) injection. Effect of diclofenac on paw withdrawal threshold in the von Frey filament test in iodoacetate-treated animals on days 3, 7 and 14 after arthritis induction. Rats were tested 45 to 60 minutes post drug. Data from12 animals/group are presented as mean ± SEM. *P < 0.05 Dunnett's test versus MIA/vehicle treated animals.

**Figure 4:**
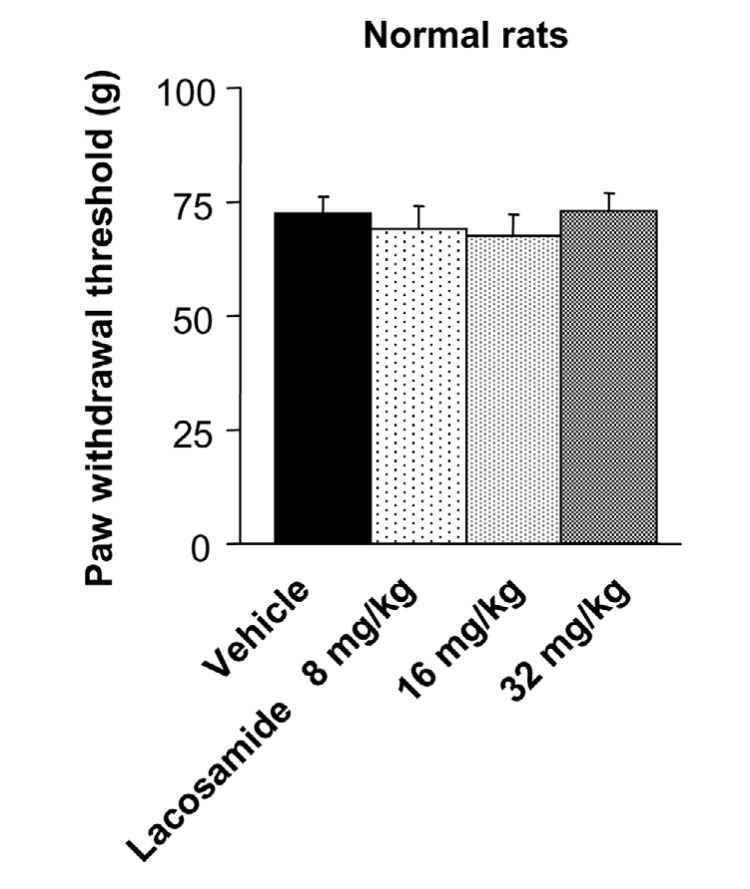
Tactile allodynia measurement in normal rats after lacosamide treatment. Effect of lacosamide on paw withdrawal threshold in the von Frey filament test in saline treated normal rats. Rats were tested 30 minutes post-drug. Data from eight animals/group are presented as mean ± SEM.

Tactile allodynia, tested with von Frey filaments, was assessed at days 3, 7, and 14 in MIA-treated rats compared to control rats (*P *< 0.05, Dunnett's test; Figures 2 and 3). Treatment with lacosamide (30 mg/kg) and morphine (3 mg) significantly improved the secondary mechanical allodynia of MIA-treated rats at days 3 and 7 but not on day 14, whereas lower doses only showed trends (*P *< 0.05, Dunnett's test; Figure 2). In the control experiment with normal rats, treatment with 8, 16, and 32 mg/kg lacosamide had no effect on pain parameters in comparison with vehicle rats (Figure 4). Diclofenac (30 mg) had no effect on secondary mechanical allodynia at all time points tested (*P *< 0.05, Dunnett's test; Figure 3).

### Effect of drug treatment on secondary mechanical hyperalgesia

**Figure 5:**
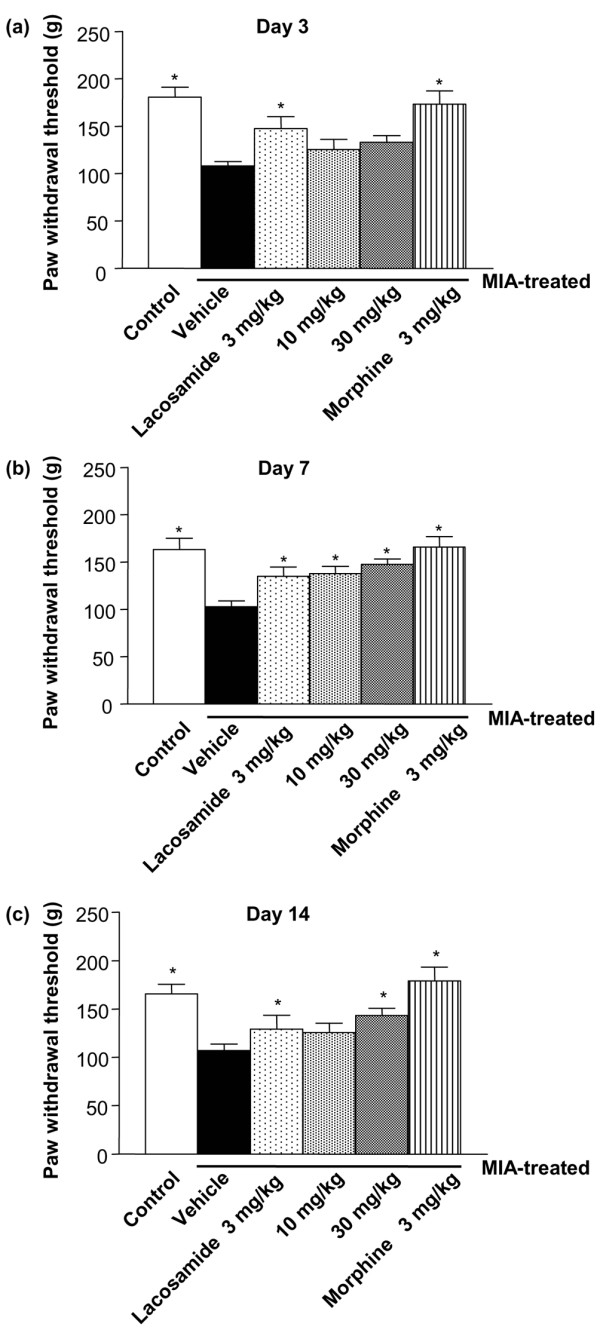
Effect of lacosamide and morphine on mechanical hyperalgesia after monosodium iodoacetate (MIA) injection. Effect of lacosamide and morphine on paw withdrawal threshold in the paw pressure test in MIA-treated animals on **(a) **day 3, **(b) **day 7 and **(c) **day 14 after arthritis induction. Rats were tested 45 to 60 minutes post-drug. Data from 12 animals/group are presented as mean ± SEM. *P < 0.05 Dunnett's test versus MIA/vehicle treated animals.

**Figure 6:**
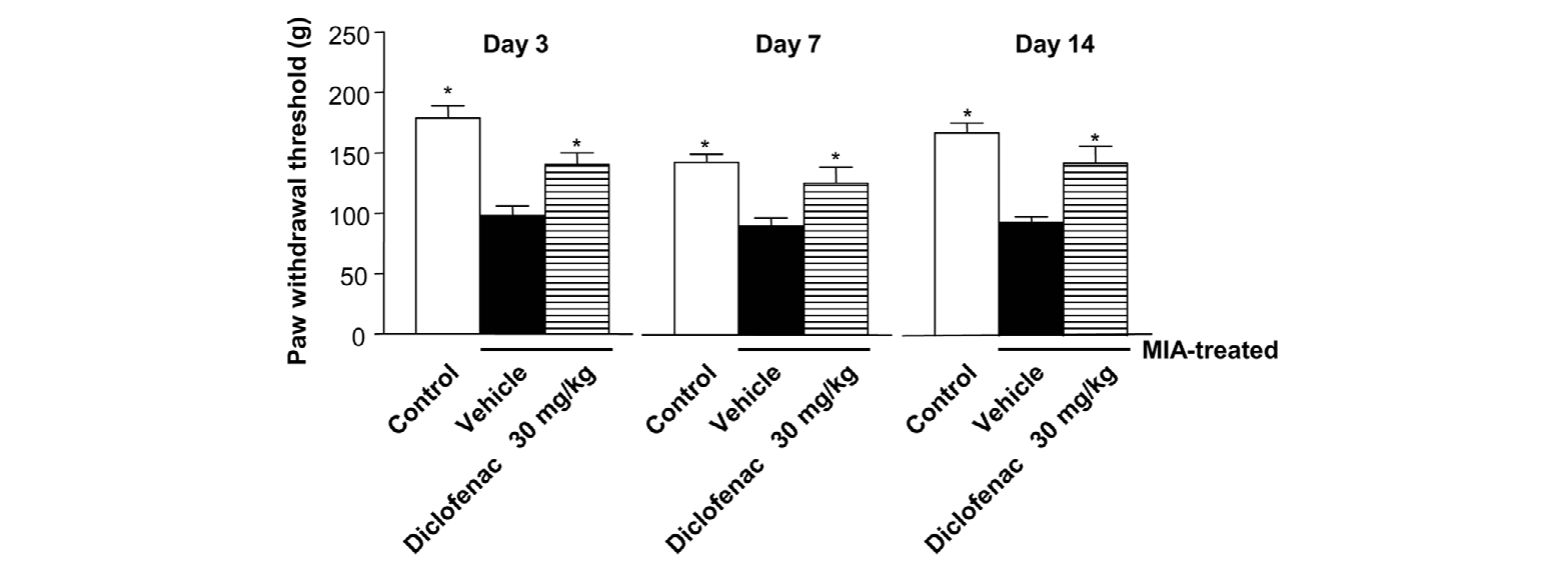
Effect of diclofenac on mechanical hyperalgesia after monosodium iodoacetate (MIA) injection. Effect of diclofenac on paw withdrawal threshold in the paw pressure test in MIA-treated animals on days 3, 7 and 14 after arthritis induction. Rats were tested 45 to 60 minutes post-drug. Data from 12 animals/group are presented as mean ± SEM. *P < 0.05 Dunnett's test versus MIA/vehicle treated animals.

There was a marked secondary mechanical hyperalgesia, as shown by a reduction in paw pressure withdrawal thresholds, in the MIA/vehicle-treated animals compared to control/vehicle treated animals (*P *< 0.05, Dunnett's test; Figures 5 and 6). Treatment of MIA-treated rats with lacosamide 3 mg/kg, diclofenac 30 mg/kg and morphine 3 mg/kg induced a significant increase (*P *< 0.05, Dunnett's test) in paw pressure withdrawal threshold compared to MIA/vehicle-treated animals on day 3. On day 7, lacosamide at all doses tested (3, 10 and 30 mg/kg), morphine and diclofenac reduced secondary mechanical hyperalgesia. The same results were seen at day 14 after MIA treatment except for the group treated with 10 mg/kg lacosamide, which did not show a significant effect (*P *< 0.05, Dunnett's test). Interestingly, in the MIA-treated animals, secondary mechanical hyperalgesia developed from day 3 on and lasted for at least 14 days compared to secondary tactile allodynia, which was more pronounced during the early phase of arthritis development, reflecting an ongoing development of pain sensitization based on different molecular mechanisms during the 14 days after MIA treatment.

## Discussion

Osteoarthritis is a chronic condition widespread in the elderly population. Patients with osteoarthritis have pain that typically worsens with weight bearing and activity. The causes of osteoarthritis have not been fully determined, although biomechanical forces affecting the articular cartilage and subchondral bone, biochemical changes in the articular cartilage and synovial membrane, and genetic factors are thought to be of importance [16]. Subchondral bone, periosteum, synovium, ligaments, and the joint capsule are all richly innervated and contain nerve endings that could be sources of nociceptive stimuli [17,18]. In addition to peripheral pain sensitization, central pain sensitization can occur in osteoarthritis [19]. Currently, no disease-modifying drugs are available so the objective of pharmacological treatment has been aimed at reducing pain, maintaining and/or improving joint mobility and reducing functional impairment.

Joint pain sensation may be caused in some states of osteoarthritis from free nerve endings in the synovium [20]. In the iodoacetate rat model for osteoarthritis the initial period implies a transient synovial inflammation. The role of macrophages in osteoarthritis has been stressed recently by Haywood and co-workers [21]. One week after MIA treatment, the inflammation is resolved in the joints and pain sensation is more likely caused by biomechanical forces affecting the articular cartilage and subchondral bone. Therefore, the iodoacetate model reflects nicely the different pain states in osteoarthritis patients.

In this study, as shown in previous publications [9,12,22], morphine (3 mg/kg) inhibited secondary tactile allodynia and mechanical hyperalgesia during the inflammatory as well as non-inflammatory period after MIA treatment. The same is true for lacosamide, which seemed to be more potent in inhibiting secondary mechanical hyperalgesia (effect at 3 to 30 mg/kg) than tactile allodynia (effect at 30 mg/kg).

Fernihough and colleagues [12] tested the anticonvulsant gabapentin (100 mg/kg) in the iodoacetate model. Gabapentin did not reduce pain behavior in the paw pressure test (mechanical hyperalgesia) but had an effect on secondary tactile allodynia at day 14 only. In the same study diclofenac, having anti-inflammatory as well as analgesic activity, was not able to reduce secondary mechanical allodynia at any time point but had an effect on mechanical hyperalgesia at all time points tested. In the present study diclofenac had no effect on secondary tactile allodynia as well but a significant reduction of mechanical hyperalgesia could be seen. The cyclooxygenase inhibitor celecoxib (30 mg/kg) was able to reduce weight bearing only after chronic treatment whereas rofecoxib (10 mg/kg) reversed alterations in weight bearing after acute treatment [8,22].

Lacosamide has also been analyzed in rat models in which pain is induced by systemic inflammation [13]. Lacosamide reversed carrageenan-induced mechanical hyperalgesia but not the carrageenan-induced acute inflammatory response (edema), suggesting that antinociception induced by lacosamide is mainly mediated by interfering with pain transmission rather than by providing anti-inflammatory effects. Lacosamide had no effect on the non-inflamed paw in doses up to 40 mg/kg. Gabapentin reversed carrageenan-induced hyperalgesia with a minimal effective dose of 10 mg/kg for mechanical hyperalgesia [23,24]. Pregabalin was active on carrageenan-induced mechanical hyperalgesia with minimal effective doses of 3 mg/kg and 6 mg/kg [23,24]. Lacosamide showed activity against Freund's complete adjuvant-induced mechanical hyperalgesia comparable to the effect of morphine [13]. In contrast, gabapentin was only weakly active against Freund's complete adjuvant-induced mechanical hyperalgesia [25,26]. Lacosamide was more potent than diclofenac on tactile allodynia and might be more potent than the anticonvulsants gabapentin and pregabalin at inhibiting mechanical hyperalgesia in arthritis rat models.

These observations indicate that the effects of drugs on disease states differ depending on their mechanisms of action and, therefore, have varying degrees of efficacy on the pathology of the tested animal model. Multiple *in vitro *pharmacological studies, for example, protein binding, functional receptor and electrophysiology studies using classical anticonvulsant targets, indicate that lacosamide does not share a common mechanism of action with other anticonvulsant drugs [27,28]. The detailed mode of action of lacosamide will be further explored. Future studies using longer term treatment with lacosamide in the iodoacetate model should identify if lacosamide has disease modifying activity in addition to its antinociceptive activity.

## Conclusion

Lacosamide had antinociceptive effects on both pain readouts assessed in the MIA model for osteoarthritis during different time points of pain development. The data presented in this publication together with recently published animal study data demonstrate lacosamide's ability to reduce arthritic pain behavior induced by multiple mechanisms. This suggests that lacosamide should be evaluated for the treatment of osteoarthritic pain in controlled clinical trails.

## Abbreviations

MIA = monosodium iodoacetate; NSAID = non-steroidal anti-inflammatory drug; SEM = standard error of the mean.

## Competing interests

BKB and TS are employees of Schwarz BioSciences GmbH, which is developing lacosamide for the treatment of diabetic neuropathic pain and epilepsy. BKB and TS are holders of patents relating to the content of this manuscript.

## Authors' contributions

NC actively participated in the experimental and organizational design of the study, developed the method for pain measurements, and carried out or supervised the experiments. NC was responsible for the evaluation of the data, as well as the statistics, and reviewed the manuscript. BKB actively participated in the experimental and organizational design of the study, gave valuable advice for the evaluation and interpretation of the experimental results, and drafted, revised, finalized, and submitted the manuscript. TS reviewed all experimental data, gave valuable advice for the evaluation and interpretation of the experimental results and the subsequent conclusions, and reviewed the manuscript.
